# A rapid appraisal of access to and utilisation of psychotropic medicines in Bihar, India

**DOI:** 10.1186/1752-4458-8-29

**Published:** 2014-07-11

**Authors:** Prianka Padmanathan, Manoj Singh, Saju C Mannarath, Mayeh Omar, Shoba Raja

**Affiliations:** 1 Faculty of Medicine and Health, University of Leeds, Worsley Building, Leeds LS2 9JT, UK; 2 Nav Bharat Jagriti Kendra, Amritnagar, Korrah, Hazaribag 825301, Jharkhand, India; 3 BasicNeeds, 114 4th Cross-, OMBR Layout, Banasawadi, Bangalore 560 043, India; 4 The Nuffield Centre for International Health & Development, Leeds Institute of Health Sciences, Charles Thackrah Building, 101 Clarendon Road, Leeds LS2 9LJ, UK

**Keywords:** Psychotropic medicines, Resource-limited settings, India, Access, Utilisation, Low- and middle-income countries, LMICS, Low-income settings

## Abstract

**Background:**

A major aspect of providing mental healthcare is access to and use of psychotropic medications. Bihar is a state in northeast India with limited mental healthcare provision; consequently access to and utilisation of psychotropic medications are likely to be limited. However, to date there has been no research assessing the situation. This study therefore aims to analyse the psychotropic medications management cycle (selection, procurement, distribution and use), and identify the barriers to access and utilisation, and their underlying causes.

**Method:**

A rapid appraisal method was used in which primary and secondary data sources were collected and analysed. Semi-structured interviews were conducted with twenty-two stakeholders and twenty-one service users from the government, non-governmental organisation (NGO) and private sectors. The qualitative data collected was analysed using a comparative thematic approach. The research was supported by the NGOs BasicNeeds and Nav Bharat Jagriti Kendra.

**Results:**

Availability, distance and cost were the main barriers to access and utilisation. At the medical college hospital level a lack of supply appears to be due to a discrepancy between orders made by the hospital and medications supplied by the manufacturers. At the primary health centre and district hospital level the main barrier is a cycle between lack of demand for treatments for mental illness by doctors and patients.

**Conclusion:**

Further investigation and monitoring is necessary to ensure the availability of psychotropic medications at the medical college hospital level. In addition, implementation of the District Mental Health Programme is likely to address the access and utilisation barriers due to its potential to break the current cycle of lack of demand.

## Background

Neuropsychiatric disorders are a major cause of disability worldwide, accounting for approximately 13% of the global burden of disease [1]. In comparison, malaria, tuberculosis and HIV/AIDS, which have been the focus of huge global initiatives, together account for only 8.2% [1]. In addition, data from the 2010 Global Burden of Disease Study demonstrated that mental and substance use disorders caused the greatest number of years lived with disability (DALYs) worldwide [2]. In low- and middle-income countries (LMICs) the burden of mental illness is rapidly increasing due to changes in demographics and epidemiology [3].

One major aspect of providing mental healthcare is the availability of and access to psychotropic medications. Worldwide it is estimated that the availability of generic medicines in the public sector is less than 60%, which results in a significant lack of access in LMICs [4]. There is a similar pattern with regard to access to essential psychotropic medications [5].

In India, a systematic review of epidemiological studies estimated the point prevalence of mental illness requiring intervention by a mental health professional to be approximately 20% [6]. These interventions can have an impact on a number of socioeconomic outcomes, by improving management of mental illness. For example a study which assessed the economic outcomes of an non-governmental organisation (NGO) programme for mental illness in Bihar and Jharkhand specifically, demonstrated an improvement in indicators such as employment status, number of days out of employment due to illness, and number of hours of care provided by a caregiver [7].

However despite mounting evidence also demonstrating the efficacy and affordability of existing medical, psychological and social interventions in low- and middle-income countries (LMICs), [8] including a number of recent studies in India, [9-11] it is estimated that 70-80% of those with mental illness in India do not receive adequate care [12]. To try to address these issues, the Indian government launched a District Mental Health Programme, which primarily aims to stimulate the development of community-based mental healthcare [13].

Bihar is the third most populous state in India with approximately 104 million people, of whom 88.7% live in rural areas [14]. Between 2009-2010 the state had the highest percentage of population living below the poverty line in India [15]. However, this percentage has fallen sharply from 53.5% to 33.74% between 2011-2012 [16]. During this period Bihar also ranked as the fastest growing state in terms of gross domestic product [17]. However, to date no district in Bihar has implemented the District Mental Health Programme. As a result there is limited mental healthcare provision within the state and access to and utilisation of psychotropic medications are likely to be limited. This research therefore aims to analyse the psychotropic medications management cycle (selection, procurement, distribution and use), and identify the barriers to access and utilisation of psychotropic medications and their underlying causes.

This research was supported by the international non-governmental organisation BasicNeeds who have formed a partnership with a local development organisation called Nav Bharat Jagriti Kendra (NBJK) to implement a community-based model for Mental Health and Development. This comprises of five interlinked modules involving capacity building, community mental health, livelihoods, research and collaboration.

## Methods

The study was carried out in five of Bihar’s thirty-eight districts: Bhojpur, Gaya, Muzaffarpur, Nalanda and Patna. The choice of districts was based on practicalities such as distance from Patna and the presence of NBJK partners who were able to facilitate the organisation of interviews locally.

A rapid appraisal method was employed, which involved the collection of data from interviews and policy and administrative documents, such as price lists and consumption data. Policy and administrative documents were obtained from stakeholders and by searching the internet.

Stakeholders were identified using a snowball sampling strategy, beginning with the programme manager for the BasicNeeds/NBJK programme. The following stakeholders were interviewed: one NBJK administrator, two government officials, one government administrator, eight psychiatrists covering six medical college hospitals (MCH) and Bihar Institute of Mental Health and Allied Sciences (BIMHAS), one medical college hospital administrator, one district hospital (DH) doctor, four primary health centre (PHC) doctors, and four community-based workers (CBW) who follow up over 900 service users between them. Three pharmacists, one hospital administrator, one government official and one government administrator declined to participate due to the requirement to sign an informed consent form. Twenty-one service users were also interviewed and were identified by CBWs or their psychiatrist. They were purposefully selected to include individuals from four districts and the private, NGO and government health sectors. The majority of these service users had experienced care from more than one sector, as they had switched between services. Interviews were semi-structured and lasted approximately twenty minutes each. Specific interview guides were developed for different types of stakeholders based on BasicNeeds’ experience in other countries. However, these questions were then developed iteratively. Interview guides are available from the corresponding author on request. All interviews were audio-recorded, although one stakeholder provided responses to interview questions in writing. Interviews in Hindi were conducted using a local interpreter.

Data collection and analysis were iterative in order to test emerging themes. A comparative thematic approach was used for the analysis of all the qualitative data. Data was transcribed and coded. Codes were grouped into themes and the themes were then analysed in relation to the research question.

Ethical approval was obtained from the University of Leeds Faculty Research Ethics Committee, UK. Considerable attempts were made to obtain ethical approval in India; however none of the ethics committees in the state agreed, due to a lack of registration to approve such a study.

## Results

**Figure 1:**
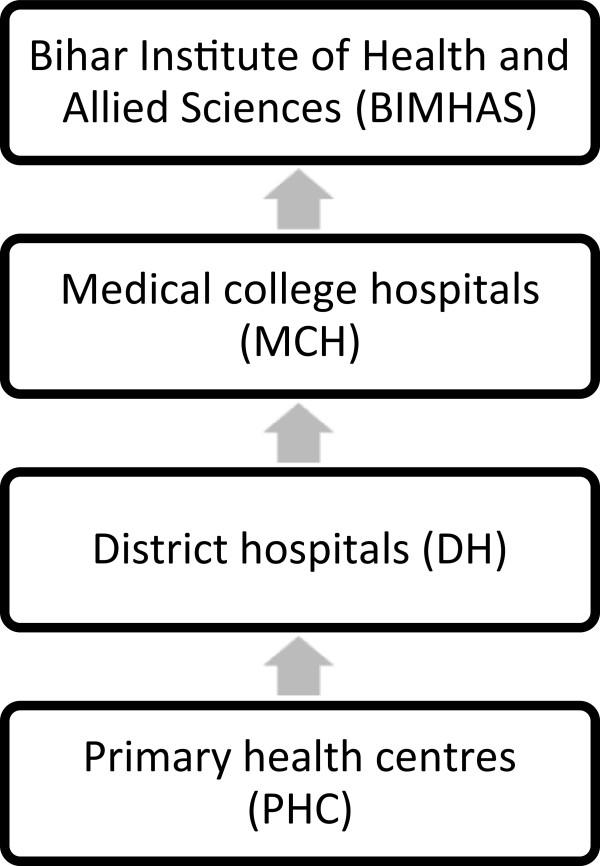
Levels of government-provided healthcare appraised in study.

Currently there are three methods of accessing evidence-based mental healthcare in Bihar: government hospitals, NGO camps, or private psychiatrists. Government mental health services are only available at medical college hospitals, and the state’s only mental health hospital, BIMHAS (Figure 1). Furthermore medications are not regularly available at the former.

Where government services are unavailable, CBWs employed by NBJK refer service users to NBJK mental health camps, which are held monthly in three districts. Approximately 400 patients attend the two largest camps. One psychiatrist is present at each camp, and the fee charged is considerably lower than those charged privately. Psychotropic medications are provided for free. Service users are then followed up in their own locality by a CBW from one of fourteen local partner NGOs.

In addition to camps, in Bihar BasicNeeds support participation in livelihood projects such as agricultural activities, trades such as carpentry and plumbing and running small businesses. They also help people with mental illness and their carers to join self-help groups and a state level user association which is involved in advocacy.

### Barriers to access and utilisation

#### ***Availability***

All ten service users currently using the NBJK camps reported that the medications they required were always available. However one service user consulting a private psychiatrist stated he previously visited the camps, but began to see the same psychiatrist privately due to a lack of availability of certain medications. Two CBWs who follow up approximately 400 service users between them, clarified that occasionally when a medication was ineffective the service user would begin consulting a psychiatrist privately, or the camp psychiatrist would prescribe an alternative that was unavailable at the camp. Therefore there were a small number of service users who had to purchase medication from private pharmacies.

The doctors at PHCs and a DH confirmed that there were no psychotropic medications available at the primary care level, except benzodiazepines which could be used to temporarily sedate patients whilst transferring them to MCHs:

“Government do not provide any psychiatric medicines except for diazepam. Diazepam is available sometimes at this PHC provided by the government as tablet and injection. Also can only prescribe diazepam for epilepsy. Other medicines we don’t have here.”

All four service users interviewed attending MCHs stated that they had to purchase the medications prescribed from private pharmacies due to lack of availability in the hospital.

Out of the three service users from BIMHAS, one stated that the medications he required were always available for free, another reported that two of the medications he was prescribed were available for free but he had to purchase the third from Patna, and the third described occasional shortages during which he had been told to return 4-5 days later or to buy the medications from private pharmacies. A CBW who follows up approximately 120 service users from BIMHAS clarified this, stating that roughly ten per cent of service users were required to purchase their medications from private pharmacies due to lack of availability. He stated that most managed to afford the medications, however a few required financial assistance from the NGO. In contrast psychiatrists working at BIMHAS stated that there were no shortages and that every patient was provided free medications.

As expected service users consulting private psychiatrists purchased psychotropic medications from private pharmacies. They reported that these medications were easily available albeit only in cities such as Patna and Gaya.

#### ***Distance***

Thirteen service users spent over an hour travelling to access treatment; six spent over 5 hours travelling. One CBW described the difficulties service users faced travelling to the camp during extreme weather:

“Medicines should be available in nearby areas because to go to the camp they have to catch the train early in the morning, but in extreme weather conditions it is difficult to collect all the patients and get them into the train. We try to get everyone here the previous night, but still some get left out and lost due to fog and it is dangerous because they are mentally ill”

Another CBW also described the difficulty of travelling a long distance when acutely mentally unwell. Service users travelling to BIMHAS were worst affected as medications were only prescribed for fifteen days, compared to a month at MCHs and camps. Service users therefore had to travel to the hospital more frequently. As the majority of the population lives in rural areas, eight service users expressed a desire for the treatments to be made available locally.

#### ***Affordability***

Out of the four service users currently attending a MCH, three described difficulties paying for medications and two had borrowed money to afford them:

“I find it expensive – I am a practitioner of homeopathy and am earning some money but that is not enough so I take money from my son.”

“Every month for about 2 or 3 days I miss the medicine. Sometimes because I cannot come to this place, sometimes because I do not have the money… I could not afford it easily. I had to take some funds from a money lender.”

Another service user who had previously attended a MCH before switching to a camp, stated that she would try to stop taking the medications as soon as she felt normal, due to the cost. A psychiatrist at one MCH stated that he referred his poorest patients to the camp, as they were unable to afford medications. He could not however, refer everyone due to a lack of capacity at the camp.

Although medications are usually available free of cost at BIMHAS and the camps, five out of the twenty-one service users struggled to pay for travel. One missed camps due to a lack of money and therefore did not take the medication regularly. Another had to borrow money, and a third had asked someone else who was attending the camp to pick up the medications on their behalf. A service user who had previously been travelling to a government hospital to consult a psychiatrist, found it more affordable to consult a local private doctor instead. This local doctor, a cardiologist, had repeated the psychiatrist’s prescription for alprazolam and olanzapine for the past two years.

One CBW stated that although the majority of service users could afford the cost, occasionally the NGO would need to provide financial assistance. Another explained that one or two per cent of service users with financial difficulties decided to revert to using superstitious remedies. In those cases the NGO tried to convince them to return to the psychiatrist and offered to pay their travel expenses for the next consultation. In contrast a third CBW stated that there were many patients who could not afford the travel expenses, therefore the NGO tried to provide them with three months of medication, to reduce the number of times they were required to attend the camp. A psychiatrist described two groups of patients: those who could not afford the cost and those who could but did not prioritise mental illness so were unwilling to pay.

### Medicines management cycle

#### ***Selection***

The World Health Organisation (WHO) regularly publishes a Model List of Essential Medicines [4]. This is a list of medications, selected on the basis of disease prevalence, safety, efficacy and cost-effectiveness, which satisfy the main healthcare needs of a population. In addition the WHO advocates the production of localised lists in order to improve supply and increase rational use.

Bihar produces a customised state level Essential Drugs List, to be used as a guideline for clinical practise and an indicator of medication availability at government facilities. The list is updated at least every two years by a core committee of experts and government officials, using similar criteria to the WHO. The published list includes an application form for the inclusion or deletion of medications. Documents outlining conflicts of interest and the specific criteria for medication selection were unavailable.

The Essential Drugs List, contains separate lists for PHCs to DHs, and the MCHs. The psychotropic medications included in the 2013-14, are shown in Table 1. In comparison to the thirteen psychotropic medications in the Essential Drugs List, the National List of Essential Medicines of India 2011 contains fifteen. The medications included are largely similar, except imipramine, olanzapine, lithium carbonate and chlorpromazine hydrochloride are included in the National List of Essential Medicines but not Bihar’s Essential Drugs List. In contrast clonazepam and trifluoperazine combined with trihexyphenidyl are included in Bihar’s Essential Drugs List but not the National List of Essential Medicines.

**Table 1:** Psychotropic medications listed in Essential Drugs List 2013 – 2014 (C-capsule; I-injection; S-syrup; SS-suspension; T-tablet)

**Primary health centres to district hospitals (out-patients)**	**Medical College Hospitals**
Alprazolam 0.25 mg, 0.5 mg (T)	Alprazolam 0.5 mg (T)
Diazepam 5 mg (T) 5 mg/ml (I)	Amitriptyline 25 mg (T)
Magnesium sulphate 500 mg/ml (I)	Carbamazepine 100 mg, 200 mg (T) 20 mg/ml (S)
	Clonazepam 0.5 mg (T)
	Diazepam 2 ml (I) (5 mg/ml)
	Fluoxetine 20 mg (C)
	Haloperidol 5 mg/ml (I)
	Lorazepam 2 mg/ml (I)
	Magnesium Sulphate 500 mg/ml (I)
	Midazolam 1 mg/ml, 5 ml (I)
	Phenobarbitone 20 mg/5 ml (SS) 200 mg/ml (I)
	Phenytoin sodium 25 mg/ml (S) 50 mg/ml (I)
	Sodium Valproate 200 mg, 500 mg (T) 200 mg/5 ml (S)
	Trifluoperazine 5 mg & Trihexyphenidyl 2 mg (T)

At BIMHAS, the state mental health hospital, the selection of medications is based on the quantity of medications distributed during the preceding 3-6 months and the perception of needs of patients by the consultant psychiatrists. Psychiatrists involved stated they were not influenced by pharmaceutical companies and kept in mind their social responsibility. The following medications were currently available: olanzapine, risperidone, lithium carbonate, sodium valproate, divalproex, clonazepam, fluoxetine, amitryptiline and imipramine. This list contains fewer medications than Bihar’s Essential Drugs List and more closely resembles the National List of Essential Medicines of India due to the inclusion of imipramine, olanzepine, lithium carbonate and exclusion of trifluoperazine combined with trihexyphenidyl. However, this was an abbreviated list that was soon to be expanded.

The local NGO, NBJK’s medicine procurement was also based on the requests of psychiatrists working at the camps, although it was stipulated that they could only request molecules rather than brands. The most recent stock list contained 21 medications, which included all those listed in Bihar’s Essential Drugs List for MCH out-patients except alprazolam and trifluoperazine combined with trihexyphenidyl.

#### ***Procurement***

**Figure 2:**
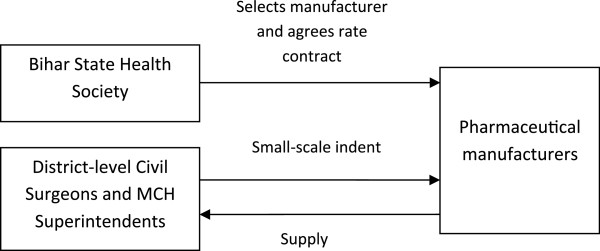
Procurement model pre-2014 (MCH: medical college hospital).

**Figure 3:**
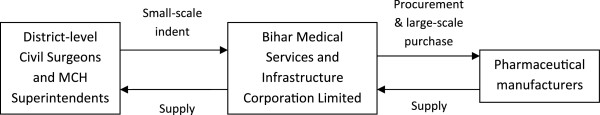
Procurement model post-2014 (MCH: medical college hospital).

The medication procurement system in Bihar is currently undergoing change. At present the Bihar State Health Society selects and approves manufacturers of medications on the Essential Drugs List centrally through a tender process. A ‘cash and carry’ model is then employed at the district level to purchase medications. An order is provided biannually by the district-level Civil Surgeon (PHCs and DH) and the Superintendent of each MCH directly to suppliers approved by Bihar State Health Society (Figure 2). The quantity ordered is based on the previous year’s consumption, disease prevalence, seasonal variation and the expected number of cases for the next six months. To minimise shortages a buffer stock of two months is maintained. PHCs and hospitals are entitled to request medication not included in the Essential Drugs List, however a tender must then be floated locally. Budget allocation to each facility is based on the expected number of patients.The new system, which is likely to be fully functional in 2014, aims to also centralise the purchasing and distribution process in order to minimise delays due to the need for pharmaceutical manufacturers to receive a payment from hospitals before supplying medications. The government has set up the Bihar Medical Services and Infrastructure Corporation Limited (BMSICL) who will procure and purchase medications at the state level. District-level Civil Surgeons and MCH Superintendents will instead order medications directly from government-owned BMSICL (Figure 3). It is yet to be decided whether a small percentage of the system will remain decentralised.

The tender process is the same for both systems. Only manufacturers of generic medications are invited. There are two stages to the process; suppliers must satisfy the technical tender before their financial bid is considered. The technical tender includes the following criteria: a minimum market standing of three years, an annual turnover over Rs 10 crore (approximately US $1.7 million), an appropriate manufacturing license, no previous black-listing. A factory inspection is also conducted in order to ensure good manufacturing practice. The rate contract is valid for one year unless specifically extended and approved suppliers are required to supply medications within 30 days of receiving an order. In addition quality is maintained by the requirement for manufacturers to submit a Test Analysis report and further testing of random and suspicious samples. If these conditions are not met, the government purchases the medications from the next level bidder, and any additional cost is recovered from the approved suppliers. Generally a minimum of 4 to 5 bids are received for each medication and Indian manufacturers are contracted.

At BIMHAS, the state mental health hospital, the medicines budget and the procurement process are currently separate to that in government hospitals, although medications are procured by the standardised process of inviting tenders. Purchases are then made every 3-6 months.

Due to the size of the organisation and the lack of a license to purchase wholesale, the NGO, NBJK procure medications by comparing quotes from three pharmacies in Jharkhand, where the organisation is based. A stock list is maintained and purchasing decisions are made monthly by a core committee based on stock levels.

#### ***Distribution***

A standard procedure is employed for all government facilities. At present the distribution channel is privatised. Medications are transported from the manufacturer to a distributer within the state and then to a wholesaler. The medications are distributed to specific facilities from the wholesaler. Under the new system, the government have purchased three regional warehouses where medications will be stored, and are in the process of setting up a transportation system. A government official acknowledged that flooding caused a challenge to distribution but generally the government would anticipate the season and procure additional medications.

Medications for the NGO camps are stored in a locked office in Bodghaya, and the expiry dates are checked monthly. They are transported by NBJK staff to each camp, often by public transport.

#### ***Use***

All the psychiatrists asked, stated that their prescribing was based on clinical experience. They believed independent guidelines were unnecessary since their training provided them with sufficient experience. Additionally they stated that pharmaceutical companies could not influence them. Instead they prescribed medications based on the patient’s financial situation. One explained,

“*…if poor patient comes to us, or to my private chamber, if I write costlier drugs, what will ultimately happen is the patient will take it for maximum 1 month, after they will stop taking the drug.”*

Table 2 provides an estimate of consumption quantity for the 2013 tender invitation, based on the previous two year’s consumption. However, one stakeholder explained that this data is unreliable:

**Table 2:** **Estimated annual consumption quantity for the year 2013-14 (Tablets and capsules unit-10x10; where different doses of the same drug were provided, estimates have been combined) **[18]

**Medication name**	**Dosage form**	**Estimated consumption quantity (10x10 tablets or capsules)**
Alprazolam	Tablet	182,563
Amitriptyline	Tablet	311,363
Carbamazepine	Tablet	76,261
Clonazepam	Tablet	57,500
Diazepam	Tablet	365,125
Diazepam	Injection	382,455
Fluoxetine	Capsules	1,585,491
Haloperidol	Injection	1000
Lorazepam	Injection	1000
Magnesium sulphate	Injection	50,845
Phenobarbitone	Suspension	1000
Phenobarbitone	Injection	1000
Phenytoin	Syrup	1000
Phenytoin	Injection	1000
Sodium Valproate	Syrup	1000
Sodium Valproate	Tablets	1000
Trifluoperazine & Trihexyphenidyl	Tablets	1000

“Frankly speaking it’s kind of data which some data entry operator has fed into the computer at sub-district level and no one knows where the data has come from. Some of the data would be genuine but it is showing wide fluctuation in some cases. I mean if I total the indent for the state, the budgeted figure is 5 times what has been spent last year.”

**Figure 4:**
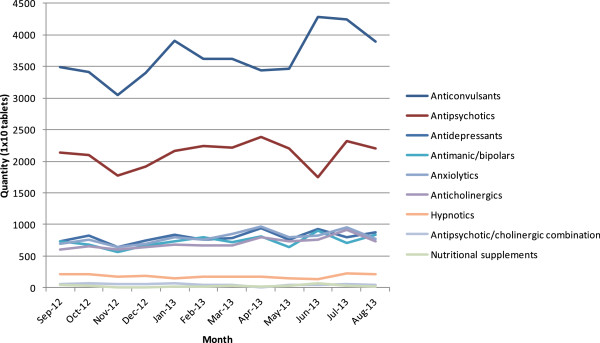
Graph showing the number of tablets consumed of different classes of psychotropic medication from September 2012 to August 2013 in NBJK camps.

**Figure 5:**
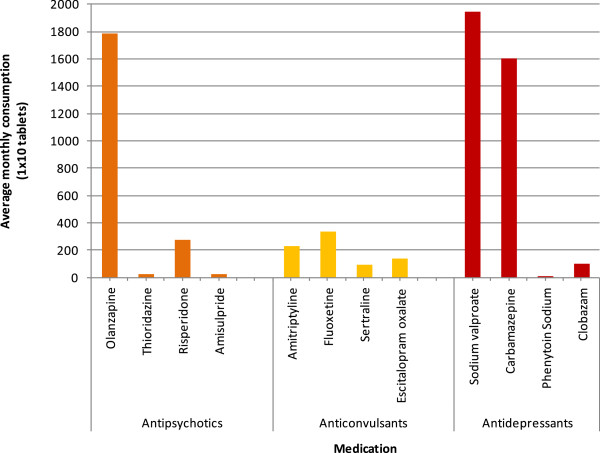
Graph showing the average monthly consumption of specific anticonvulsant, antipsychotic and antidepressant medications between September 2012 and August 2013 in NBJK camps.

The Bihar Medical Services and Infrastructure Corporation Limited are therefore aiming to rectify this by closely monitoring consumption over time, using a new electronic system.Figure 4 shows the consumption trends for different classes of psychotropic medications over one year in NBJK camps.Anticonvulsants are the most commonly prescribed medications followed by antipsychotics and antidepressants. A further breakdown of the data indicates that sodium valproate and carbamazepine are the two most frequently prescribed anticonvulsants (Figure 5). This only partially reflects the government consumption data in which carbamazepine consumption appears to greatly exceed the other anticonvulsants. In terms of antipsychotics, olanzapine is the most commonly prescribed at the NBJK camps. The prescribing of antidepressants appears to be more variable.

### Underlying causes of barriers to access and utilisation

A government official believed the lack of availability of psychotropic medications in PHCs and DHs, and their limited inclusion in the Essential Drugs List for these levels, was due to a lack of demand. PHC doctors confirmed this stating that very few people with mental illness attended the clinics. However, they explained that this was due to their lack of facilities and medication to treat mental illness. One stated,

“…they know there is no facility or doctor in this hospital or these types of hospitals; they directly go to Patna or somewhere else.”

This view was reiterated by a stakeholder at Bihar Medical Services and Infrastructure Corporation Limited, who stated that consumption figures are highly dependent on availability. Therefore the corporation expects that once the new procurement system is fully functional, and previous problems with supply are addressed, consumption figures will increase. It is expected that the annual medicines budget, which in 2013 was roughly 200 crore (approximately US $33 million), will increase to over 300 crore (approximately US $49 million) due to increased demand.

Within PHCs however, opinion varied about treating mental illness. One PHC doctor stated that training of PHC doctors was necessary, whilst another felt that psychiatry was “super-specialised” and so they required a psychiatric doctor. This was supported by a psychiatrist who stated,

“*It* (psychotropic medications) *should not be freely available at PHC level. It should be made available only where a specialist psychiatrist is stationed as part of the medical team, otherwise it is likely to be misused.*”

In addition, the PHC doctors were unable to name any psychotropic medications that they would like to be made available. However they also described the need for a “social mobiliser” or counsellor, in addition to medications.

In MCHs, where psychiatrists were present but psychotropic medications were usually unavailable, one psychiatrist speculated that poor availability may be due to lack of awareness and sensitivity at the level of decision makers. However a MCH administrator stated that they had sufficient funds to purchase the medications they required, including any essential medications that the psychiatric department requested. The administrator and the Head of Department at this MCH stated that although orders were sent to suppliers for psychotropic medications, the medications were often not supplied. This problem was less common amongst other medications therefore they believed it was due to the low volume purchased, both by the hospital and throughout the state. The low prices negotiated through the tender process, meant that supplying psychotropic medications was only profitable for the pharmaceutical company if large volumes were purchased.

“There is a vicious circle, two three years back the State Health Society has approved the rates, and some of the companies, the rates are so low they are unable to supply…If I give an order of carbamazepine 10,000 tablets, if it is not in bulk the company will not supply.”

#### ***Limitations***

This study aimed to analyse the psychotropic medicines management cycle (selection, procurement, distribution and use), and identify the access and utilisation barriers of psychotropic medicines and their underlying causes. The research was limited by the lack of pharmacists willing to participate. As a result it was not possible to determine their role in the distribution system. As pharmacists are likely to be a key stakeholder, it is important that their role is explored further to gain a more comprehensive understanding of the distribution system and to identify the source and reliability of consumption data.

In addition the effect of the researcher, interpreter and NGO staff must be considered. For example the presence of a member of the NGO staff during interviews may have resulted in response bias. However, confidentiality was emphasised and efforts were made to ensure service users in particular did not feel pressurised to respond in a certain way.

The snowball sampling strategy may have led to sampling bias and limited representativeness, particularly as it began with the NGO programme manager. This was minimised by ensuring that stakeholders from both the private and government healthcare sector were also included and involved in identifying further stakeholders.

Finally the inclusion of only five districts may have also limited the representativeness of this study. However as these were central districts in which camps and BIMHAS were situated, it is likely that the results underestimate the significance of barriers such as cost and distance.

## Discussion

Despite these limitations this study raises a number of points for discussion. Interviews of service users and CBWs indicate that psychotropic medications are available in the NGO and private sector. However, cost and distance are the main barriers to access. The findings suggest that these barriers could lead to non-adherence to treatment, and therefore may reduce the effectiveness of such treatment. They can also necessitate compromises in prescribing safety, such as the provision of large quantities of medications to last several months and psychiatric consultations without the service user being present.

In terms of medicines management, the inclusion of psychotropic medication in the Essential Drugs List is promising. However, the lack of availability in MCHs needs to be explored further. Comparison is required between the quantity of psychotropic medications ordered by Heads of Departments and Superintendents and the quantity supplied. Close monitoring of supply is especially necessary to investigate the hypothesis that medications are being ordered but not supplied. If this is due to the low quantity of medications ordered by each hospital, the problem may be addressed when Bihar Medical Services and Infrastructure Corporation Limited begin purchasing medications in bulk. However, if it is due to the low volume of medications required by the state in total, this would add further weight to the scaling up of mental healthcare.

In addition, the reason for the differences between Bihar’s Essential Drugs List and the medications provided at the state mental health hospital, BIMHAS needs to be explored. For example it is unclear why trifluoperazine combined with trihexyphenidyl is one of two anti-psychotics listed in the Essential Drugs List for MCHs, when consumption appears to be low and it has not been selected for BIMHAS or the National List of Essential Medicines.

The current government medicines management system appears to be well organised, and the new centralised procurement system has the potential to bring about significant improvements. In addition the mandatory testing of stock is beneficial in ensuring quality is upheld, whilst the maintenance of a buffer stock and additional procurement before rainy seasons demonstrates good insight. The difference between the government and NGO procurement, storage and distribution processes highlights the efficiency savings and other benefits gained by large-scale state provision of medications.

**Figure 6:**
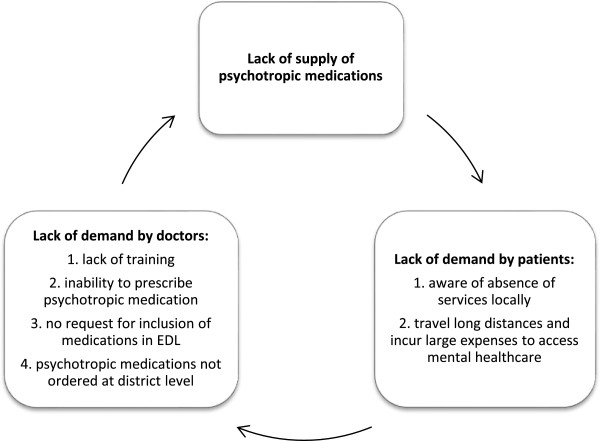
Cycle of lack of demand by doctors and service users at the primary health centre and district hospital level (EDL: Essential Drugs List).

The consumption data from NBJK camps provides a valuable insight into prescribing, since current government consumption data is unreliable. It is particularly interesting that antipsychotics, used for severe mental disorders, are more commonly prescribed than antidepressants which are indicated for common mental disorders. This may partially reflect the frequent prescription of the antipsychotic, olanzapine for sleeping difficulties, despite not being an indication for its use in the National Formulary of India [19]. This irrational use can lead to harm and therefore would require further investigation. More importantly it may also suggest that people with common mental disorders are not accessing treatment.Finally, this report highlights the cycle between lack of demand for treatments for mental illness at the PHC and district level by doctors and patients, which is the main barrier to access and use of psychotropic medications (Figure 6).

Implementation of the revised District Mental Health Programme has the potential to break this cycle: the presence of staff trained to treat mental illness using both pharmacological and psychosocial interventions will lead to people accessing mental healthcare locally, which will in turn result in demand for, and supply of, psychotropic medications. Most importantly this will address the barriers of availability, cost and distance. However, for implementation to be a success, resistance amongst stakeholders to less qualified healthcare staff being permitted to prescribe must be addressed.

## Competing interests

Financial competing interests: none to declare. Non-financial competing interests: BasicNeeds and Nav Bharat Jagriti Kendra are non-governmental organisations which advocate for improvements to mental healthcare.

## Authors’ contributions

PP prepared the study protocol, obtained funding and ethical approval, collected and analysed the data and drafted the manuscript. MS coordinated the data collection and was involved in revising the manuscript. SM helped with attempts to obtain ethical approval in India, participated in designing the study and was involved in revising the manuscript. MO helped to obtain ethical approval in the United Kingdom and was involved in revising the manuscript. SR conceived and helped design the study and was involved in revising the manuscript. All authors have read and approved the final manuscript.
